# Low tension recruits the yeast Aurora B protein Ipl1 to centromeres in metaphase

**DOI:** 10.1242/jcs.261416

**Published:** 2023-08-17

**Authors:** Heather D. Edgerton, Soumya Mukherjee, Marnie Johansson, Jeff Bachant, Melissa K. Gardner, Duncan J. Clarke

**Affiliations:** ^1^ Department of Genetics, Cell Biology & Development, University of Minnesota, Minneapolis, MN 55455, USA; ^2^ Department of Molecular Cell Systems Biology, University of California, Riverside, Riverside, CA 92521, USA

**Keywords:** Ipl1, Aurora B, Bub1, Top2, Centromere tension

## Abstract

Accurate genome segregation in mitosis requires that all chromosomes are bioriented on the spindle. Cells monitor biorientation by sensing tension across sister centromeres. Chromosomes that are not bioriented have low centromere tension, which allows Aurora B (yeast Ipl1) to perform error correction that locally loosens kinetochore–microtubule attachments to allow detachment of microtubules and fresh attempts at achieving biorientation. However, it is not known whether low tension recruits Aurora B to centromeres or, alternatively, whether low tension directly activates Aurora B already localized at centromeres. In this work, we experimentally induced low tension in metaphase *Saccharomyces cerevisiae* yeast cells, then monitored Ipl1 localization. We find low tension recruits Ipl1 to centromeres. Furthermore, low tension-induced Ipl1 recruitment depended on Bub1, which is known to provide a binding site for Ipl1. In contrast, Top2, which can also recruit Ipl1 to centromeres, was not required. Our results demonstrate cells are sensitive to low tension at centromeres and respond by actively recruiting Ip1l for error correction.

## Teaser

**Summary:** Yeast Aurora B (Ipl1) is recruited to metaphase centromeres under low tension, dependent on Bub1.

## INTRODUCTION

Faithful segregation of the genome in mitosis depends on a collaboration between complex mechanical processes and regulatory checkpoint controls, which together facilitate chromosome biorientation before anaphase onset. Both occupancy of kinetochore attachment sites by microtubules and the tension between sister kinetochores are monitored by cells. A lack of stable kinetochore occupancy and/or low tension delay mitotic progression from metaphase to anaphase via the spindle assembly checkpoint (SAC) (Jia et al., 2013; Khodjakov and Pines, 2010; Maresca and Salmon, 2010; Pinsky and Biggins, 2005; Tauchman et al., 2015). Recent studies in mammalian cells have highlighted the importance of tension sensing. Although kinetochore occupancy appears to be established very quickly in early prometaphase, cells do not initiate anaphase until ∼40 min later, when all chromosomes are aligned at the metaphase plate and kinetochore tension is high (Sikirzhytski et al., 2018). Moreover, initial occupancy can be achieved by a mesh of non-centrosomal microtubules or by syntelic or merotelic attachments that do not generate high tension. Therefore, occupancy and high tension are unlikely to be established at the same time, and it seems that high tension is required to trigger silencing of the SAC to permit anaphase onset.

When proper biorientation is not achieved, cells are thought to sense the resulting low tension at centromeric (herein also described as CEN) regions. This capability is consistent with the tension between properly bioriented attachments being much higher than the tension imposed on kinetochores due to thermal forces (Chacon et al., 2014; Ye et al., 2016). Upon sensing low tension, an error correction mechanism is activated, mediated by Aurora B kinase, which resides at the inner centromere regions (Cheeseman et al., 2006; Cimini et al., 2006; Jia et al., 2013; Pinsky et al., 2006; Welburn et al., 2010). This acts locally to facilitate microtubule detachment from kinetochores under low tension, allowing another chance at achieving proper biorientation to provide high tension between sister centromeres. Once an Aurora B-dependent detachment event occurs, the lack of occupancy at that kinetochore activates the canonical Mad2-dependent SAC to block anaphase onset until biorientation of that chromosome has been achieved. In the context of low tension, Aurora B localization at centromere regions is required for error correction and SAC activation.

Properly bioriented chromosomes are subjected to enough tension to substantially alter the distances between the two sister centromeres as well as the distance between the outer kinetochore and the inner kinetochore within a sister (Maresca and Salmon, 2009; Nannas and Murray, 2014; Stephens et al., 2013; Suzuki et al., 2016, 2011; Uchida et al., 2009; Warsi et al., 2008). Under low tension, a decrease in these distances is thought to promote access of Aurora B to its substrates to weaken the kinetochore–microtubule interface and promote detachment events. Although this tension-sensing mechanism is not fully understood, the initial concentration of Aurora B at centromere regions seems to be central to error correction when tension is low (Lampson and Cheeseman, 2011).

There are several known pathways that recruit Aurora B to centromeres/kinetochores and recruitment occurs as part of the chromosome passenger complex (CPC) (Broad and DeLuca, 2020; Broad et al., 2020; Klein et al., 2006). At inner centromeres and kinetochore-proximal centromere regions, the CPC bridges the interaction of Aurora B with centromere specific phosphorylation of histone H3 and H2A, established by the haspin and Bub1 kinases, respectively (Broad and DeLuca, 2020; Broad et al., 2020; Carmena et al., 2012; Jeyaprakash et al., 2011; Kelly et al., 2010; Peplowska et al., 2014; Sawicka and Seiser, 2014). Haspin kinase phosphorylates H3 threonine 3 (H3T3), providing a binding site for the survivin CPC component (Dai and Higgins, 2005; Jeyaprakash et al., 2011; Kelly et al., 2010; Wang et al., 2010). Evidence from studies in yeast and in *Xenopus* egg extracts support a model where haspin is recruited by direct interaction with SUMO2- or 3-modified topoisomerase II at centromeres to promote H3T3 phosphorylation (Edgerton et al., 2016; Yoshida et al., 2016). Bub1 kinase phosphorylates H2A threonine 120 (H2A Serine 121 in yeast) at kinetochore-proximal centromeres, which recruits shugoshin (Sgo1), providing a binding site for the borealin CPC component (Kawashima et al., 2007; Peplowska et al., 2014; Tsukahara et al., 2010; Yamagishi et al., 2010). In addition to these interactions, other studies have shown CPC–Aurora B or Ipl1 recruitment to inner kinetochores via the COMA complex (Cairo et al., 2023; Fischbock-Halwachs et al., 2019; Garcia-Rodriguez et al., 2019), and even to spindle microtubules (Campbell and Desai, 2013). Despite the detailed information about these recruitment mechanisms, the biological contexts that induce Aurora B recruitment to centromeres remain less well understood.

As in higher eukaryotes, the budding yeast Aurora B ortholog Ipl1 is recruited to inner centromeres when chromosomes are undergoing the process of biorientation (Adams et al., 2000; Ainsztein et al., 1998; Biggins and Murray, 2001; Gassmann et al., 2004; Kaitna et al., 2000; Nozawa et al., 2010; Pinsky et al., 2006, 2003). Thus, upon kinetochore capture by spindle microtubules, cells are primed for activation of Ipl1 at inner centromeres under conditions of low tension, such as when erroneous attachments are formed (Biggins and Murray, 2001; Stern and Murray, 2001). Although the binding sites of Ipl1 at inner centromeres are conserved with those of Aurora B in vertebrates (as described above), whether Aurora B and Ipl1 recruitment is stimulated by low tension is not well understood. In this work, we used metaphase arrest in yeast cells to experimentally induce the loss of Ipl1 from centromeres. Then, we induced low tension to test whether Ipl1 was specifically recruited under low-tension conditions. The data provide evidence that active recruitment of Ipl1 is indeed induced under low-tension conditions. SUMOylation of the C-terminal domain of Top2 was dispensable for Ipl1 recruitment under conditions of low tension, whereas Bub1 was essential. Thus, recruitment of Ipl1 likely occurs via Sgo1 binding to H2AT121p and not through the H3T3p pathway. The data provide direct evidence for the proposed surveillance mechanism that is responsive to low tension and reacts by facilitating Ipl1 recruitment to centromeres to facilitate error correction (Biggins and Murray, 2001; Cimini et al., 2006; Hauf et al., 2003; Kallio et al., 2002; Lampson and Cheeseman, 2011; Lampson et al., 2004; Pinsky et al., 2006; Stern and Murray, 2001).

## RESULTS

### Ipl1 is evicted from centromeres under high tension in arrested metaphase cells

**Fig. 1.**
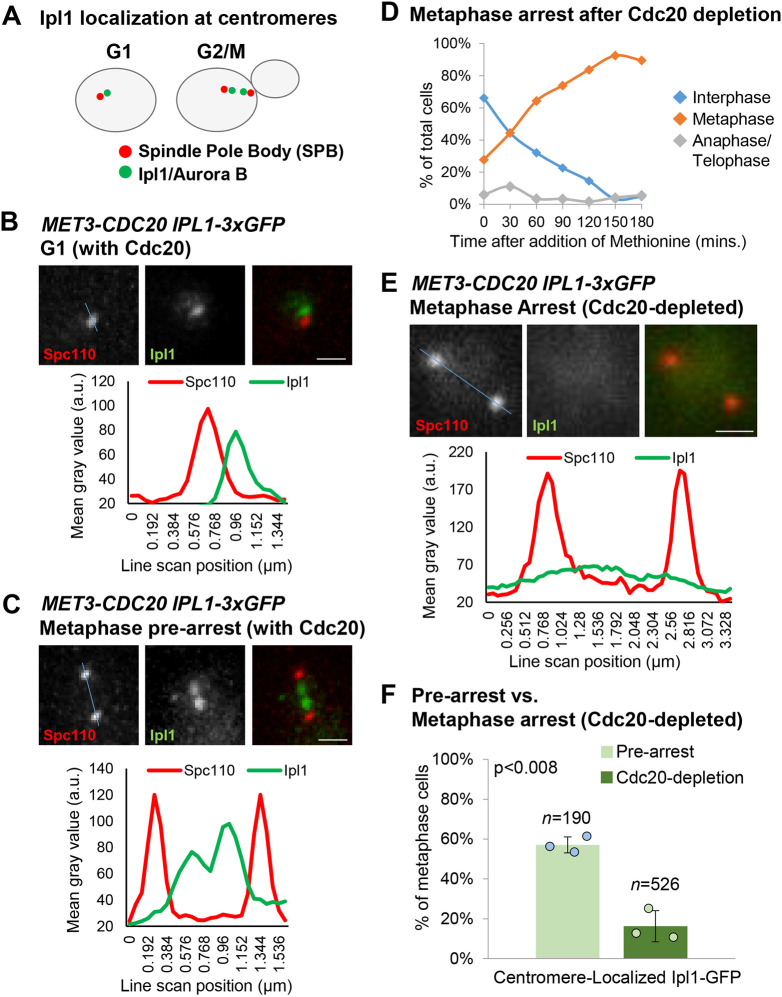
**Ipl1 is displaced from centromeres in Cdc20-depleted metaphase-arrested cells.** (A) Cartoon showing the known localization of Ipl1 (green) relative to the positions of the spindle poles, SPBs (red), at the clustered centromeres in budding yeast G1 and G2/M (metaphase) cells. In metaphase, the centromeres lie on the spindle axis. (B,C) Representative images and quantification of Ipl1 localization (line scans across the blue line in the images) in G1 and metaphase cells, relative to the positions of the SPBs. (D) Accumulation of cells in metaphase following Cdc20 depletion after addition of methionine to *MET3-CDC20* cells. (E) Representative image and quantification of Ipl1 localization after metaphase arrest induced by Cdc20 depletion. (F) Classification of metaphase cells based on whether Ipl1–GFP is localized at centromeres (as in C) or is diffusely localized (as in E). The values plotted are means of at least three experimental repeats (*n*=total number of cells analyzed), individual experiment means shown by the filled circles. Error bars are s.d. *P*-values calculated using paired one-tailed *t*-tests. Scale bars: 1 µm. a.u., arbitrary units.

In yeast cells, kinetochores are assembled soon after centromere replication in S-phase. These nascent sister kinetochores rapidly recruit Ipl1 to their inner centromere regions, concomitant with the assembly of the spindle apparatus and the process of chromosome biorientation (Fig. 1A) (Biggins and Murray, 2001; Pinsky et al., 2006, 2003). Although the process of biorientation coincides with Ipl1 recruitment, it is not known whether this initial recruitment of Ipl1 is dependent on low-tension at centromeres ahead of biorientation. To explore this question, we employed Cdc20 depletion, a well-characterized experimental strategy to arrest cells in metaphase with bioriented chromosomes under high-tension (Campbell and Desai, 2013; Keating et al., 2009; Mukherjee et al., 2019; Nerusheva et al., 2014). Anaphase initiation is induced by the APC/C ubiquitin ligase. Placing the APC/C-activating subunit, Cdc20, under control of the *MET3* promoter allows depletion of Cdc20 upon addition of methionine to growth medium, which then causes cells to accumulate in metaphase (Keating et al., 2009). Importantly, previous studies have measured the high tension under these conditions and quantified kinetochore detachments, revealing that they are rare (Mukherjee et al., 2019). In *MET3-CDC20* cells, we first examined endogenous Ipl1 tagged with GFP before Cdc20 depletion. In G1, Ipl1–GFP localized as expected to a clustered focus of centromeres close to the single spindle pole body (SPB; visualized via Spc110–mCherrry) (Fig. 1B). Ipl1–GFP also localized as previously reported to the pair of clustered sister centromere foci in cells with assembled metaphase spindles (G2/M cells) (Fig. 1C).

Next, we characterized metaphase arrest upon depletion of Cdc20. Within 2.5 h of methionine addition, the percentage of metaphase cells increased from ∼28% to ∼93% (Fig. 1D). Strikingly, the cohort of metaphase-arrested cells had a dramatic decrease in the percentage of cells with Ipl1–GFP localized at centromeres, consistent with previous reports (Campbell and Desai, 2013; Nerusheva et al., 2014) and with the prediction that prolonged high-tension induces the eviction of Ipl1 from inner centromeres (Fig. 1E,F). This suggests that the maintenance of Ipl1 occupancy at centromeres is sensitive to centromere tension.

### Ipl1 is recruited to centromeres in arrested metaphase cells upon depletion of Cin8

**Fig. 2.**
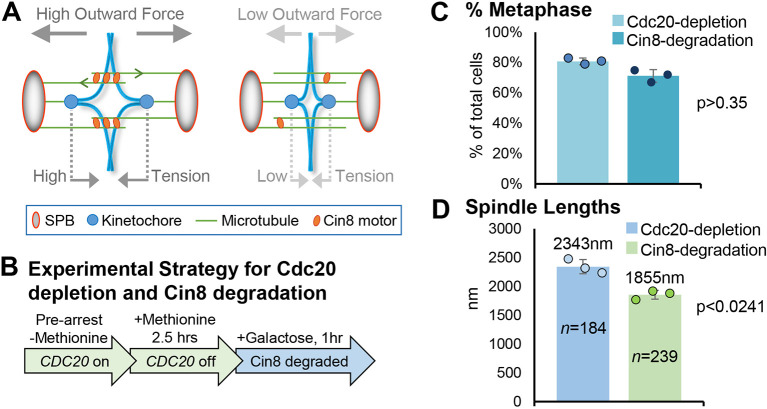
**Cdc20-depletion-mediated metaphase-arrested cells have reduced spindle length following Cin8 degradation.** (A) Cartoon depicting the forces on sister centromeres with (left) and without (right) the Cin8 outwardly directed motor protein activity. Overlap microtubules (green) are slid apart by Cin8 motors (orange) generating outward force on the SPBs (ovals), which in turn leads to high tension on centromere regions (blue circles) via the attached kinetochore microtubules. Without Cin8 (right) mitotic spindles become shorter without the outward force resulting in low tension on centromeres. (B) Experimental scheme used to arrest cells in metaphase by Cdc20 depletion and induce low tension after Cin8 degradation (Mukherjee et al., 2019). (C) Percentage of metaphase cells after Cdc20 deletion and then after Cin8 degradation. Cdc20-depletion, 2.5 h after addition of methionine; Cin8-degradation, 2.5 h with methionine, then 1 h with galactose. (D) Metaphase spindle lengths in arrested Cdc20-depleted cells and then after Cin8 degradation, based on measuring the distances between SPBs. The values plotted are means of at least three experimental repeats (*n*=total number of cells analyzed), individual experiment means shown by the filled circles. Error bars are s.d. *P*-values calculated using paired one-tailed *t*-tests.

Without Cdc20, cells arrest in metaphase because the ultimate target of the SAC, securin (yeast Pds1), cannot be degraded and thus anaphase cannot be initiated. However, the data described above reveal that Ipl1 leaves inner centromeres under these conditions, presumably because biorientation of the chromosomes has been achieved and each pair of centromeres is under prolonged high tension. Given that Ipl1 leaves centromeres under high tension in metaphase-arrested cells, we sought to test whether lowering the tension would reinstate Ipl1 to centromeres. Because detachments are rare under these conditions (Mukherjee et al., 2019), we reasoned that we could examine the direct effects of low tension without the complication of co-existing detached centromeres. Moreover, because Ipl1 was evicted from centromeres during the initial metaphase arrest, and given that detachment requires Ipl1 to phosphorylate its kinetochore substrates under these conditions (Mukherjee et al., 2019), re-recruitment of Ipl1 would need to occur before error correction could be initiated. Therefore, we ought to be able to distinguish the effects of low centromere tension versus kinetochore detachments under these conditions and ask whether Ipl1 is recruited to centromere regions *de novo* if conditions arise that result in low tension. Low tension can be induced in metaphase, after chromosome biorientation, by depleting the outward force generating kinesin-5 motor protein Cin8 (Hildebrandt and Hoyt, 2000; Mukherjee et al., 2019). The outward force generated by Cin8 is due to sliding apart of overlapping antiparallel interpolar microtubules, thus pushing SPBs away from each other, which acts to increase the tension on centromeres via the kinetochore microtubules (Fig. 2A). Cin8 depletion in metaphase reduces centromere tension and then induces detachment events where individual kinetochores detach from their microtubule (Mukherjee et al., 2019). To deplete Cin8, we used the previously characterized strategy where endogenous *CIN8* is replaced with a degron-tagged *CIN8* allele in the *MET3-CDC20* cells (Fig. 2B) (Mukherjee et al., 2019).

**Fig. 3.**
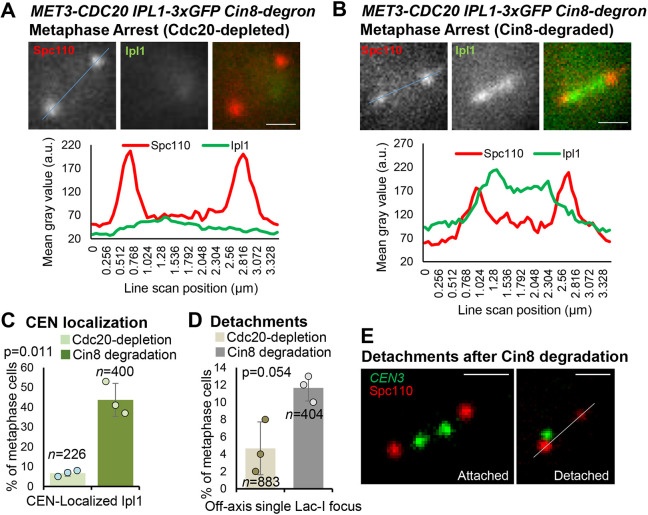
**Ipl1 is recruited to centromeres upon Cin8 degradation in metaphase-arrested cells.** (A,B) Representative images and quantification of Ipl1 localization (line scans across the blue line in the images) before and after induction of low tension (i.e. after Cdc20 depletion then Cin8 degradation). (C) Classification of metaphase cells based on whether Ipl1–GFP is localized at centromeres or is diffusely localized. (D,E) Representative images and quantification of kinetochore detachment events, as described in the main text. The gray line in the image on the right shows the spindle axis and indicates that the coalesced sister centromeres of chromosome III (the diffraction-limited spot, green) have been displaced off the axis. The values plotted are means of at least three experimental repeats (*n*=total number of cells analyzed), individual experiment means shown by the filled circles. Error bars are s.d. *P*-values calculated using paired one-tailed *t*-tests. Scale bars: 1 µm. a.u., arbitrary units.

First, cells were arrested in metaphase following depletion of Cdc20, as described in Fig. 1. Then, addition of galactose induced expression of the *UBR1* ubiquitin ligase, which initiates degron-Cin8 degradation (Fig. 2B). Samples were collected at *t*=0, immediately after addition of galactose, and at *t*=1 h to evaluate Ipl1–GFP localization both before and after Cin8 degradation. Previous studies have found the timing of these shifts in carbon source to be optimal when using this experimental strategy (Mukherjee et al., 2019). Here, we observed efficient metaphase arrest after Cdc20 depletion, as in Fig. 1, and that the percentages of cells arrested in metaphase at both *t*=0 and *t*=1 h were similar (Fig. 2C). This indicates that most cells remained arrested during the 1 h galactose incubation. Previous studies (Mukherjee et al., 2019) have shown that this experimental scheme induces Cin8 degradation within 1 h and that an immediate consequence is a reduction in spindle length, due to the reduced outward forces, accompanied by low tension at centromeres (Fig. 2A, right). To confirm this, we measured spindle lengths at *t*=0 and *t*=1 h, revealing that, as expected, there was a reduction of ∼20.8% in mean spindle length after the galactose incubation, which induces Cin8 depletion and low tension (Fig. 2D). These data are therefore consistent with results from previous reports that measured tension directly after Cin8 degradation (Mukherjee et al., 2019). At *t*=0, in the metaphase arrested Cdc20-depleted cells, there was a low frequency of cells with Ipl1 localized at centromeres (Fig. 3A–C), consistent with the same conditions in Fig. 1E,F. In contrast, at *t*=1 h, in the arrested metaphase cells with reduced spindle length following Cin8 depletion, there was a striking recruitment of Ipl1–GFP to centromere regions (Fig. 3B,C).

Finally, if low tension after Cin8 depletion recruited active Ipl1 to centromeres, we would expect to observe detachment of microtubules from kinetochores, as has been previously reported (Kapoor et al., 2000; O'Connell et al., 2008), because detachment events in low-tension cells requires Ipl1 (Biggins and Murray, 2001; Mukherjee et al., 2019; Pinsky et al., 2006, 2003). To measure detachments, we used identical experimental conditions, metaphase arrest induced by Cdc20 depletion followed by low tension induced by Cin8 degradation, but instead of observing Ipl1–GFP, we used strains in which the centromere of chromosome III (*CEN3*) is marked by LacI–GFP (via integrated LacO repeats; Straight et al., 1996) (Fig. 3D,E). This allows detachments to be identified because loss of the microtubule from one sister kinetochore results in coalescence of the sister centromere LacI–GFP signals into a single diffraction limited spot as well as loss of the positioning on the spindle axis (between the SPBs). A representative example is shown in Fig. 3E, right. After the induction of Cin8 degradation, there was a ∼2.5-fold increase in detachments, consistent with Ipl1 recruitment to centromeres activating the error correction mechanism that weakens the binding of microtubules to kinetochores under low tension (Fig. 3D,E) (Miller et al., 2016). This increase in detachments is consistent with previous studies using similar experimental conditions (Mukherjee et al., 2019). We also note that the fold increase in detachments is likely an underestimate because: (1) to be conservative, we excluded coalesced LacI–GFP foci that remained on the spindle axis (i.e. these were categorized as ‘attached’), and (2) because only one out of the sixteen yeast chromosomes was being observed. Altogether, the evidence supports that low tension induced in arrested metaphase cells actively mediates recruitment of Ipl1 to centromere regions to promote biorientation through error correction.

### Ipl1 is recruited to centromeres in arrested metaphase cells upon treatment with benomyl

**Fig. 4.**
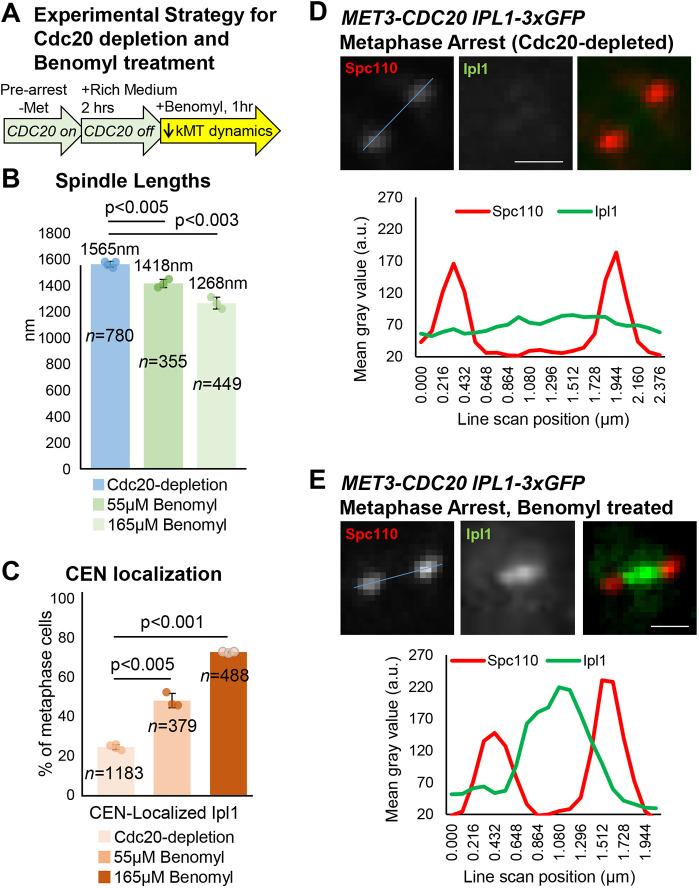
**Ipl1 is recruited to centromeres in metaphase-arrested cells following benomyl treatment.** (A) Experimental scheme used to arrest cells in metaphase by Cdc20 depletion and treatment with benomyl. (B) Metaphase spindle lengths in the arrested Cdc20-depleted cells and then after benomyl treatment, based on measuring the distances between SPBs. (C) Classification of arrested metaphase cells based on whether Ipl1–GFP is localized at centromeres or is diffusely localized, before and after 1 h benomyl treatment. (D,E) Representative images and quantification of Ipl1 localization (line scans across the blue line in the images) before and after benomyl treatment in Cdc20-depleted metaphase arrested cells. The values plotted are means of at least three experimental repeats (*n*=total number of cells analyzed), individual experiment means shown by the filled circles. Error bars are s.d. *P*-values calculated using paired one-tailed *t*-tests. Scale bars: 1 µm. a.u., arbitrary units.

An alternative method of reducing centromere tension in metaphase-arrested cells is treatment with benomyl. Several reports have provided evidence that benomyl concentrations that suppress kinetochore microtubule dynamics can do so without inducing detachments and yet reduce centromere tension (Marco et al., 2013; Pearson et al., 2006; Suzuki et al., 2016). Using the same experimental set-up described above, we arrested cells by Cdc20 depletion then treated them with benomyl for 1 h before imaging Ipl1–3xGFP (Fig. 4A). Consistent with reduced centromere tension, 55–165 µM benomyl reduced spindle length by ∼10–19% (Fig. 4B). Benomyl also increased the frequency of metaphase-arrested cells with Ipl1 localized between the SPBs (Fig. 4C–E). Therefore, these data provide further evidence that induction of low tension in metaphase-arrested cells results in Ipl1 recruitment to centromeres.

### Low tension-induced recruitment of Ipl1 does not require SUMOylation of the topoisomerase II C-terminal domain

In yeast and vertebrates, Ipl1/Aurora B is recruited to centromeres as a component of the CPC. Several pathways have been described that facilitate CPC recruitment via interaction with centromere-specific nucleosomes (see Introduction). In yeast, one pathway requires SUMOylation of the topoisomerase II (Top2) C-terminal domain (CTD) that promotes the recruitment of haspin kinase to centromeres where it phosphorylates histone H3 threonine 3 (H3T3p), providing a direct binding site for CPC. To test whether this pathway is required for Ipl1 recruitment to centromeres induced by low tension, we generated *MET3-CDC20 degron-CIN8* strains (as used above) and possessing *top2* mutant alleles, either lacking the entire CTD (*top2ΔCTD*) or lacking the conserved CTD SUMOylation sites (*top2-SNM*; SNM is a mnemonic for ‘SUMO no more’ Bachant et al., 2002).

**Fig. 5.**
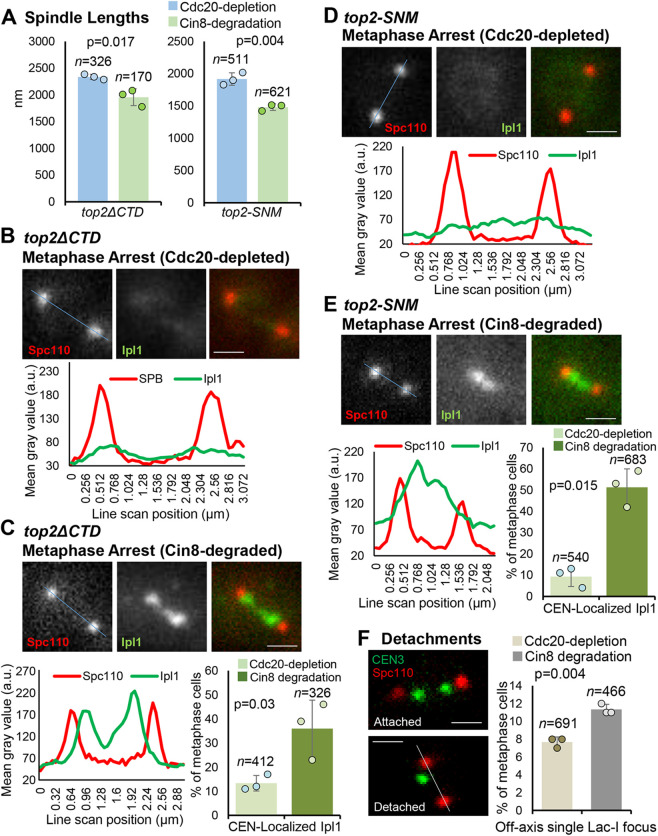
**Ipl1 is recruited to centromeres after Cin8 degradation in metaphase-arrested *top2-SNM* and *top2ΔCTD* mutants.** (A) Metaphase spindle lengths in arrested Cdc20-depleted cells and then after Cin8 degradation, based on measuring the distances between SPBs, in the *top2* mutants. (B–E) Representative images and quantification of Ipl1 localization (line scans across the blue line in the images) before and after induction of low tension (i.e. after Cdc20 depletion then Cin8 degradation) in the *top2* mutants. Graphs show the classification of metaphase cells based on whether Ipl1-GFP is localized at centromeres or is diffusely localized. (F) Representative images and quantification of kinetochore detachment events, as described in the main text. The gray line in the lower image follows the spindle axis and indicates that the coalesced sister centromeres of chromosome III (the diffraction-limited spot, green) have been displaced off the axis. The values plotted are means of at least three experimental repeats (*n*=total number of cells analyzed), individual experiment means shown by the filled circles. Error bars are s.d. *P*-values calculated using paired one-tailed *t*-tests. Scale bars: 1 µm. a.u., arbitrary units.

Consistent with the *TOP2* wild-type strain analyzed in Figs 2 and 3, metaphase arrest followed by depletion of Cin8 resulted in a decrease in spindle length in both the *top2ΔCTD* and the *top2-SNM* strains (Fig. 5A). Similarly, upon metaphase arrest, few cells had centromeric Ipl1–GFP (Fig. 5B–E). However, after induction of Cin8 degradation, Ipl1–GFP was recruited to centromeres, similar to the results with the Top2 wild-type strain. These experiments demonstrate that SUMOylation of Top2 as well as the entire CTD of Top2 are dispensable for recruitment of Ipl1 to centromeres under conditions of low tension. Consistent with Ipl1 recruitment under low tension not requiring SUMOylation of Top2, the *top2-SNM* mutant had an increase in detachments after Cin8 depletion (Fig. 5F), like the wild-type *TOP2* strain (Fig. 3D,E). Altogether, the data indicate that Top2 SUMOylation is dispensable for Ipl1 recruitment in arrested metaphase cells as well as for the activation of error correction by Ipl1.

### Bub1 kinase is required for low tension-dependent recruitment of Ipl1 to centromeres

**Fig. 6.**
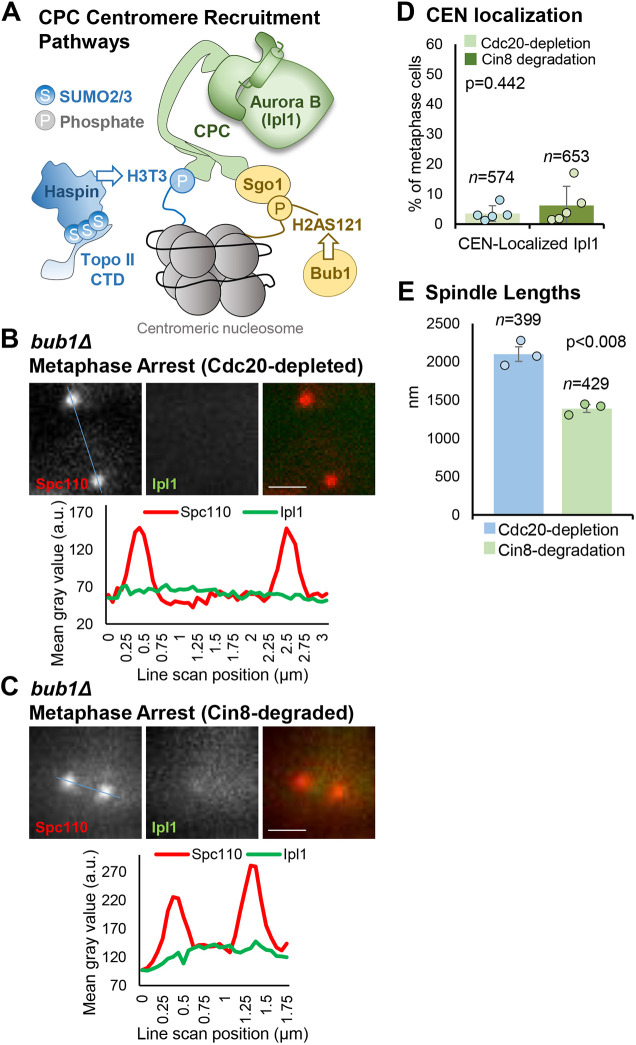
**Ipl1 is not recruited to centromeres after Cin8 degradation in metaphase arrested *bub1Δ* cells.** (A) Cartoon depicting two known mechanisms that can recruit Ipl1/Aurora B to centromeres in mitosis. lpl1/Aurora B binds to centromeres as a component of the CPC complex (green), which interacts with nucleosomes (gray) via centromere-specific histone modifications: H3T3p and H2AS121p (via Sgo1). The centromere-specific histone modifications are established by haspin kinase (recruited to centromeres by the SUMOylated Topo II C-terminal domain; CTD) and Bub1 kinase. (B,C) Representative images and quantification of Ipl1 localization (line scans across the blue line in the images) before and after induction of low tension (i.e. after Cdc20 depletion then Cin8 degradation) in the *bub1Δ* cells. (D) Classification of metaphase cells based on whether Ipl1–GFP is localized at centromeres or is diffusely localized in *bub1Δ* cells. (E) Metaphase spindle lengths in arrested Cdc20-depleted cells and then after Cin8 degradation, based on measuring the distances between SPBs, in the *bub1Δ* cells. The values plotted are means of at least three experimental repeats (*n*=total number of cells analyzed), individual experiment means shown by the filled circles. Error bars are s.d. *P-*values calculated using paired one-tailed *t*-tests. Scale bars: 1 µm. a.u., arbitrary units.

Top2 SUMOylation and the Top2 CTD are not required for low tension-dependent recruitment of Ipl1 to centromeres in arrested metaphase cells. Another major mechanism capable of recruiting CPC to inner centromeres depends on Bub1 kinase, which in yeast is mediated by phosphorylation of histone H2A serine 121 at centromeres by Bub1 (Fig. 6A). The interaction of CPC with H2AS121p occurs via the bridging molecule Sgo1 (Fig. 6A) and Bub1 inactivation delocalizes Sgo1 from centromeres (Liu et al., 2013; Peplowska et al., 2014). Therefore, to test whether Sgo1 is required for Ipl1 recruitment under low tension, we crossed the *MET3*-*CDC20*, *CIN8-degron, IPL1-GFP* strain to a *sgo1Δ* strain. However, no segregants could be recovered with the desired allele combination, indicating that *sgo1Δ* is synthetically lethal with one or a combination of the *MET3*-*CDC20*, *CIN8-degron* or *IPL1-GFP* alleles.

As an alternative approach to deleting *SGO1*, we generated *MET3*-*CDC20*, *CIN8-degron*, *IPL1-GFP* strains with *BUB1* deleted. Strains lacking Bub1 have been previously characterized and are known to be unable to generate the H2AS121p nucleosomes that Sgo1 binds to (Kawashima et al., 2010). This combination was compatible with viability, allowing us to examine metaphase-arrested and Cin8-depleted cells. Like the wild-type and *top2* mutant strains (Figs 3 and 5), there were few Cdc20-depleted metaphase-arrested cells with Ipl1–GFP localized at centromeres, once more indicating that prolonged arrest with bioriented chromosomes under high tension results in delocalization of Ipl1–GFP (Fig. 6B–D). After addition of galactose to induce Cin8 degradation, there was no increase in cells with centromeric Ipl1–GFP, revealing that Bub1 is indeed required for recruitment of Ipl1–GFP to centromeres under conditions of low tension (Fig. 6B–D). Spindle lengths were decreased after Cin8 depletion, relative to the Cdc20-depleted metaphase-arrested cells, consistent with a similar reduction in centromere tension compared to the wild-type and *top2* mutant strains (Fig. 6E). Together, the data provide evidence that prolonged high tension at centromere regions leads to delocalization of Ipl1 from centromeres, and that the induction of low tension activates a Bub1-dependent mechanism to recruit Ipl1 back to centromeres.

## DISCUSSION

Directly evaluating the effects of low centromere tension is complicated because low tension promotes error correction by Aurora B which leads to detachment of microtubules from kinetochores. This means it is problematic to investigate direct consequences of low centromere tension versus low microtubule occupancy. Here, we have attempted to circumvent this using an experimental set-up where detachments are rare, by arresting cells in metaphase with centromeres under high tension following Cdc20 depletion in yeast. Criteria for distinguishing low-tension centromeres from detachment events have been described in detail previously using this experimental set-up (Mukherjee et al., 2019; Parmar et al., 2023). Those studies quantified detachments after Cdc20 depletion-induced metaphase arrest. Very few cells had detachment events under these conditions (Mukherjee et al., 2019; Parmar et al., 2023), consistent with the data presented here. Moreover, during the induced metaphase arrest, Ipl1/Aurora B becomes dispersed from centromeres, as shown by our data as well as two previous studies (Campbell and Desai, 2013; Nerusheva et al., 2014). Almost all cells lacked detectable Ipl1 at the CEN regions following the metaphase Cdc20 arrest. If detachments were prevalent during this induced metaphase arrest, then Ipl1 would be seen to localize to the CENs, but that was not the case. This is consistent with detachments being rare. Following the induced metaphase arrest, Cin8 was then degraded in the arrested metaphase cells. Mukherjee et al. measured centromere tension using this experimental set-up and quantified the reduction in tension following Cin8 degradation, as well as showing that subsequent detachment events are dependent on Ipl1 (Mukherjee et al., 2019). Therefore, together with the data presented here, the evidence indicates that detachment is induced by Ipl1 re-localized to the CENs, and that Ipl1 re-recruitment is a consequence of Cin8 degradation and must precede detachment events.

We cannot rule out that rare detachment events in some arrested metaphase cells induce recruitment of Ipl1 via the well-established SAC pathway, independently of low tension. However, in these uncommon cases, Ipl1 would be recruited specifically to the unattached CEN in each individual cell and would be seen as an individual small discrete focus of Ipl1. That is, Ipl1 would not be re-recruited to all the CENs at once. This is based on evidence showing that kinetochores respond autonomously when detached (e.g. Salimian et al., 2011). In contrast, the Ipl1 signals we observe after Cin8 degradation are large foci of fluorescence, presumably representing recruitment to the majority, if not all, CENs. This is consistent with Cin8 degradation affecting the entire spindle, which in turn reduces tension at all CENs (see Fig. 2A).

The kinesin-5 motor protein Cin8 promotes sliding apart of overlap microtubules at the spindle equator and this generates outward force to generate CEN tension. Depleting Cin8 therefore directly reduces centromere tension (Mukherjee et al., 2019). However, another study has shown that Cin8 can recruit Glc7 (the yeast PP1 phosphatase) to kinetochores (Suzuki et al., 2018) and it has been shown that Glc7 opposes Ipl1 activity by dephosphorylating Ipl1 substrates at kinetochores. An important question in the context of our experiments, is whether CPC-Ipl1 was recruited to centromeres due to low tension, or whether a lack of Glc7 at kinetochores indirectly induced recruitment of CPC-Ipl1. Our experiments might provide evidence to distinguish these two roles of Cin8. The Cdc20 depletion (2.5 h) and metaphase arrest removes Ipl1 from CENs (Campbell and Desai, 2013; Nerusheva et al., 2014; this work). This likely provides a substantial time window for Cin8 to bring PP1 to kinetochores where it ought to thoroughly act on kinetochore substrates (opposed by Ipl1), thus allowing high tension via stabilization of the microtubule–kinetochore interface. Based on published work using Cdc20 depletion to arrest cells in metaphase, this involves PP2A-dependent dissociation of Sgo1 (and Bub1) from CENs which leads to dissociation of Ipl1 (Nerusheva et al., 2014). In our experiments, when we then induced Cin8 degradation, Glc7 presumably no longer gets to the kinetochores (Suzuki et al., 2018), but Ipl1 is not concentrated there under the Cdc20 arrest conditions and so any re-phosphorylation of Ipl1 substrates (opposed by Glc7) is likely to be inefficient. Meanwhile, the lack of Cin8 causes SPBs to come closer together because the Cin8 outward force is abolished, and CEN tension is directly reduced across the whole spindle (Mukherjee et al., 2019; Parmar et al., 2023). We propose it is low tension that recruits Ipl1 back to CENs via Bub1. At that point, it can be envisaged that the lack of Glc7 at CENs contributes to detachment events via weakened microtubule–kinetochore interfaces, as the re-recruited Ipl1 phosphorylates its kinetochore substrates.

Previous studies have revealed that there are several mechanisms that can recruit CPC-Aurora B or -Ipl1 to centromeres, kinetochores and mitotic spindles (Bonner et al., 2019; Broad and DeLuca, 2020; Broad et al., 2020; Cairo et al., 2023; Campbell and Desai, 2013; Fischbock-Halwachs et al., 2019; Garcia-Rodriguez et al., 2019; Haase et al., 2017; Hadders et al., 2020; Liang et al., 2020; Marsoner et al., 2022). In our experiments, we did not attempt to distinguish between these different localization patterns of Ipl1, and instead we defined recruitment as concentrated localization between the SPBs. Although this is a shortcoming of our approach, we did determine that Ipl1 recruitment was dependent on Bub1, which indicates recruitment was likely to be to the centromere-proximal kinetochore region. We cannot, however, rule out that some Ipl1 is recruited to other regions between the SPBs including spindle microtubules. This is important to note because Ipl1 highly concentrated on metaphase spindles is functional for the essential mitotic functions of Ipl1 (Campbell and Desai, 2013). Furthermore, published data are consistent with the COMA complex recruiting Ipl1 to inner kinetochores and Bub1 bringing it to centromere-proximal kinetochore regions, and both mechanisms seem to be sufficient for accurate chromosome segregation in mitosis. Our data suggest that the COMA-mediated recruitment pathway is unable to recruit Ipl1 under our experimental conditions following Cin8 depletion given that *bub1*-null mutants could not re-recruit Ipl1.

A pivotal previous study in mammalian cells provided evidence that CPC-Aurora B localization at centromeres is sensitive to chromosome alignment status (Salimian et al., 2011). They examined cells 45 min or 1 h after release from monastrol treatment, which arrests cells in early mitosis with mono-polar spindles. During the initial arrest, CPC-Aurora B became localized at centromeres as it does in an unperturbed early mitosis; that is, coincident with chromosome capture (Carmena et al., 2012). Following the monastrol wash-out, when the cells had almost fully formed metaphase plates and presumably most chromosomes were properly bioriented, Salimian et al. analyzed chromosomes lying off the plate (misaligned chromosomes). They found that in these cells Aurora B levels at centromeres were higher on the misaligned chromosomes than on chromosomes at the metaphase plate. Importantly, live-cell imaging of Aurora B revealed a tight positive correlation between misalignment and high centromeric Aurora B in a dynamic manner. This shows that centromeres are responsive to alignment in terms of Aurora B association. These studies are consistent with low tension inducing CPC-Aurora B recruitment dynamically, similar to what we found in our studies using yeast cells, although it is not known if the misaligned chromosomes analyzed by Salimian et al. were under low tension or could have had detached kinetochores. Of particular interest is that Salimian et al. found higher levels of H2A-T120p on the misaligned chromosomes they examined, whereas H3T3p levels were unaltered. This is consistent with our yeast data showing that Bub1 is required for Ipl1 re-recruitment upon Cin8 degradation and reduction in tension in metaphase.

A significant finding is that Ipl1-dependent detachments occur following Ipl1 re-recruitment under the experimental conditions used here (i.e. after Cin8 degradation) (Mukherjee et al., 2019). This indicates that re-recruitment of Ipl1 activates error correction to weaken microtubule–kinetochore interfaces and facilitate microtubule detachment under low tension. This has been previously shown to be due to phosphorylation of key Ipl1 substrates at kinetochores (Asbury et al., 2006; Cheeseman et al., 2001; DeLuca et al., 2006; Gestaut et al., 2008; Lampert et al., 2010; Tien et al., 2010; Welburn et al., 2010). In addition, the requirement for Bub1 is consistent with recent work demonstrating that low tension can activate the spindle checkpoint via Bub1 even in the absence of microtubule detachments (Proudfoot et al., 2019). Therefore, activation of Bub1 as an early step when tension is low prevents anaphase onset and provides time for error correction to be induced. The evidence suggests that Bub1 provides central functions under conditions of low centromere tension.

Altogether, previous studies and the data presented here are consistent with a current model for how cells achieve accurate chromosome segregation, by detecting erroneous microtubule attachments via sensing low tension at centromere regions. According to this model, cells respond by activating the spindle checkpoint and error correction via Bub1 and Aurora B/Ipl1.

## MATERIALS AND METHODS

### Yeast strains and growth conditions for live-cell imaging

Yeast strains (made available on request) were derivatives of W303 (Table S1). Strains with Ipl1-3xGFP or CEN3:lacO×33 and GFP-lacI were originally provided by Trisha Davis (University of Washington, Seattle, WA, USA) then crossed to generate the stains used. Strains harboring *top2-SNM* and *top2ΔCTD*, replacing *TOP2* at the endogenous locus, were previously described (Edgerton et al., 2016). Strains containing *MET3-CDC20* and *degron-CIN8* were described previously (Mukherjee et al., 2019). All stains were grown overnight in synthetic yeast growth medium lacking methionine (USBiological) to maintain *CDC20* expression. *Degron-CIN8* strains were grown in the above medium with 4% raffinose, whereas other strains were grown in the above medium with 2% dextrose. For fluorescence microscopy, medium was supplemented with additional adenine (200 mg/l). Overnight cultures were diluted to an optical density at 600 nm (OD) of 0.15–0.3 and cultured for 4 h, before addition of 20 mg/l methionine to deplete Cdc20, and where stated, for another 1 h with 2% galactose to induce Cin8 degradation. Before live cell microscopy, yeast cells were washed and cultured in a microfluidic chamber as described previously (Chacon et al., 2014; Edgerton et al., 2016). All strains (except those in the benomyl treatments) were imaged by TIRF microscopy with an Eclipse-Ti microscope (Nikon) using sapphire lasers (Coherent) (488 nm and 561 nm) to visualize GFP and mCherry at 30°C. Rapid switching achieved near-simultaneous imaging between red and green lasers and images captured with an iXon3 EMCCD camera (Andor Tech.) and CFI Apochromat 100×, 1.49-NA objective (Nikon). For the benomyl treatment experiments in Fig. 4, cells were imaged using a DeltaVision Ultra microscope fitted with an Olympus 60×/1.42, Plan Apo N objective (UIS2, 1-U2B933) and PCO-Edge sCMOS camera (>82% QE). Entire cell volumes were obtained by capturing 6.5 µm thick Z-series with 0.2 µm spacing. Z-series were deconvolved then projected using SoftWoRx software. All images were contrast enhanced using ImageJ (Fiji).

### Image analyses

Ipl1 localization in cells was categorized as previously described Edgerton et al. (2016). Briefly, images were binned into the following categories (e.g. see Fig. 1C,E): (1) centromeric (CEN), where the GFP signal was concentrated in foci clustered within the spindle axis and the peak gray value was similar to the peak values of the SPBs; (2) diffuse, where the GFP signal was homogenous and low intensity relative to the SPBs. For determining the percentage of metaphase cells in the population, DIC microscopy and imaging of the SPBs was performed using a Zeiss AxioPlan 2 microscope. Cells were classified as metaphase if the SPBs were greater than 1 µm apart. Cells were classified as interphase where a bud was absent or if small-budded cells had either a single SPB or if the SPBs were less than 1 µm apart. Anaphase/telophase cells had SPBs separated across the bud neck and were at least 4 µm apart. Spindle lengths were measured in ImageJ (Fiji) using the line tool or using custom code written in MATLAB (available upon request) to identify spindle pole bodies then calculate the distance between poles. In the case of the latter, the poles were identified by thresholding-based image segmentation and using the centroid function to identify the center of the poles. To identify cells with a detachment event, SPBs and the sister centromeres of chromosome III were imaged. The chromosome III centromeres were observed indirectly using strains with tandem LacO repeats integrated adjacent to the centromere and expressing LacI–GFP, as previously described (Mukherjee et al., 2019). Detachment events had to conform to two criteria with the LacI–GFP spots: (1) being coalesced, and (2) lying off the spindle axis (see Figs 3E and 5F). Spindle axes were determined using a straight line connecting the center of each SPB. We note that this method results in a conservative estimate of the frequency of detachments as some detached chromosomes can lie within the spindle axis. Nevertheless, this avoids overestimating detachments because coalesced centromere spots can be observed in metaphase due to normal centromere dynamics.

### Statistical analysis

To determine the statistical significance of differences in Ipl1 inner centromere localization, spindle length differences, and differences in the percentage of cells with detachment events between strains, paired one-tailed *t-*tests were performed using the means of at least three independent experimental repeats. *P*-values and total numbers of cells analyzed are given in each plot.

## Supplementary Material

10.1242/joces.261416_sup1 Supplementary information
