# Supplementary material for: Low tension recruits the yeast Aurora B protein Ipl1 to centromeres in metaphase
Source: J Cell Sci. 2023 Aug 17;136(16):jcs261416. doi: 10.1242/jcs.261416 (PMC10445749; doi:10.1242/jcs.261416)
Supplement: Supplementary information [file joces-136-261416-s1.pdf]

Table S1. Yeast strains used in this study.

MATα cdc20::pMET3-CDC20(TRP1) IPL1-3xGFP(HIS3) SPC110::SPC110-mCherry(hphMX)
MATa cdc20::pMET3-CDC20(TRP1) IPL1-3xGFP(HIS3) SPC110::SPC110-mCherry(hphMX) ura3::pCUP1-Ndegron-CIN8(URA3) his3::GAL-UBR1-MYC(HIS3)
MATa CEN3:LacOx33(Kan) his3::GFP-lacI(HIS3) SPC110::SPC110-mCherry(hphMX) cdc20::pMET3-CDC20(TRP1) ura3::pCUP1-Ndegron-CIN8(URA3) his3::GAL-UBR1-MYC(HIS3)
MATa cdc20::pMET3-CDC20::TRP1 SPC110::SPC110-mCherry(hphMX) IPL1-3xGFP(HIS3) GAL-UBR1-MYC::HIS3 pCUP1-Ndegron-CIN8::URA3 top2::Kan leu2::MET3-Ub-DHFR-HA-TOP2(LEU2) top2-deltaCTD(TRP1)
MATα cdc20::pMET3-CDC20(TRP1) IPL1-3xGFP(HIS3) SPC110::SPC110-mCherry(hphMX) ura3::pCUP1-Ndegron-CIN8(URA3) his3::GAL-UBR1-MYC(HIS3) top2-SNM-HA(Kan)
MATa CEN3:LacOx33(Kan) his3::GFP-lacI(HIS3) SPC110::SPC110-mCherry(hphMX) ura3::pCUP1-Ndegron-CIN8(URA3) his3::GAL-UBR1-MYC(HIS3) top2-SNM-HA(Kan)
MATα cdc20::pMET3-CDC20(TRP1) IPL1-3xGFP(HIS3) SPC110::SPC110-mCherry(hphMX) ura3::pCUP1-Ndegron-CIN8(URA3) his3::GAL-UBR1-MYC(HIS3) bub1::KAN

Journal of Cell Science • Supplementary information
